# Incidental Detection of Carcinoma In-situ in Fibroadenoma of the Breast in a Young Woman: A Rare Finding

**DOI:** 10.7759/cureus.3797

**Published:** 2018-12-31

**Authors:** Atul Tiwari, Brij Mohan K Singh, Shubham Varshney, Mohan L Yadav

**Affiliations:** 1 Department of Pathology, Mahatma Gandhi Medical College & Hospital, Jaipur, IND; 2 Department of Pathology, Kasturba Medical College, Manipal, IND

**Keywords:** fibroadenoma, carcinoma in situ, ductal carcinoma in situ

## Abstract

Fibroadenoma, being a very common benign tumor of the breast in young females, does not pose any threat and, thus, can be treated with lumpectomy. Breast cancer arising within a fibroadenoma is a rare phenomenon, but detecting these neoplasms is of utmost importance for complete treatment and follow-up. These lesions are an incidental finding in a lumpectomy specimen done for fibroadenoma breast.

A 28-year-old female presented with multiple bilateral lumps for eight years. The lumps were mobile, non-tender, and slowly growing, with no nipple discharge, no axillary lymphadenopathy, and no family history. The diagnosis of a benign lesion suggestive of fibroadenoma was given on ultrasonography (USG) and fine-needle aspiration cytology (FNAC). The patient underwent lumpectomy and the excised tissues were subjected to histopathological examination. Grossly, multiple, well-circumscribed, encapsulated masses, with the largest measuring 4x2.5x2 cm were noted. All masses showed homogenous grey-white areas with slit-like spaces. On microscopy, predominant areas were consistent with fibroadenoma, with few foci showing the features of ductal carcinoma in situ (DCIS) with nests of cells having pleomorphic nuclei, prominent nucleoli with atypical mitosis, a cribriform pattern, and comedo necrosis.

This highlights the rare association of fibroadenoma and carcinoma in situ, thus, a careful and extensive sampling of the tissue is required to prevent the false negative diagnosis by pathologists.

## Introduction

A fibroadenoma, being a very common benign tumor of the breast in young females, does not pose any threat and, thus, can be treated with lumpectomy [1]. Breast cancer arising within fibroadenoma is a rare phenomenon, but detecting these neoplasms is of utmost importance for complete treatment and follow-up. These lesions are an incidental finding in a lumpectomy specimen done for fibroadenoma breast [2].

## Case presentation

**Figure 1:**
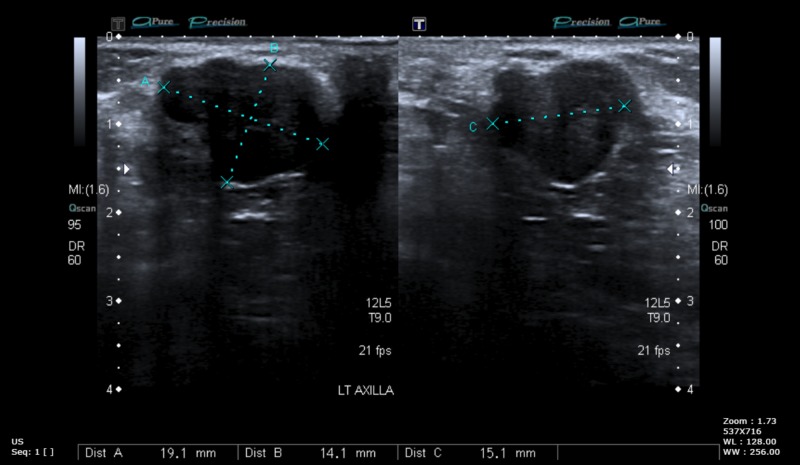
USG: Hypoechoic lesion USG: ultrasonography

A 28-year-old female presented with multiple bilateral breast lumps for eight years. The lumps were mobile, non-tender, and slowly growing. On examination, multiple freely mobile lumps in both breasts (eight on the right side, four on the left side) with well-defined margins, firm consistency, and a smooth surface were identified. There was no tenderness or local rise in temperature, no history of ulcers, puckering, dimpling, or swelling. They were not associated with any change during the menstrual cycle. There was no family history of breast carcinoma. Ultrasonography (USG) showed multiple hypoechoic lesions suggestive of fibroadenoma (Figure 1).

**Figure 2:**
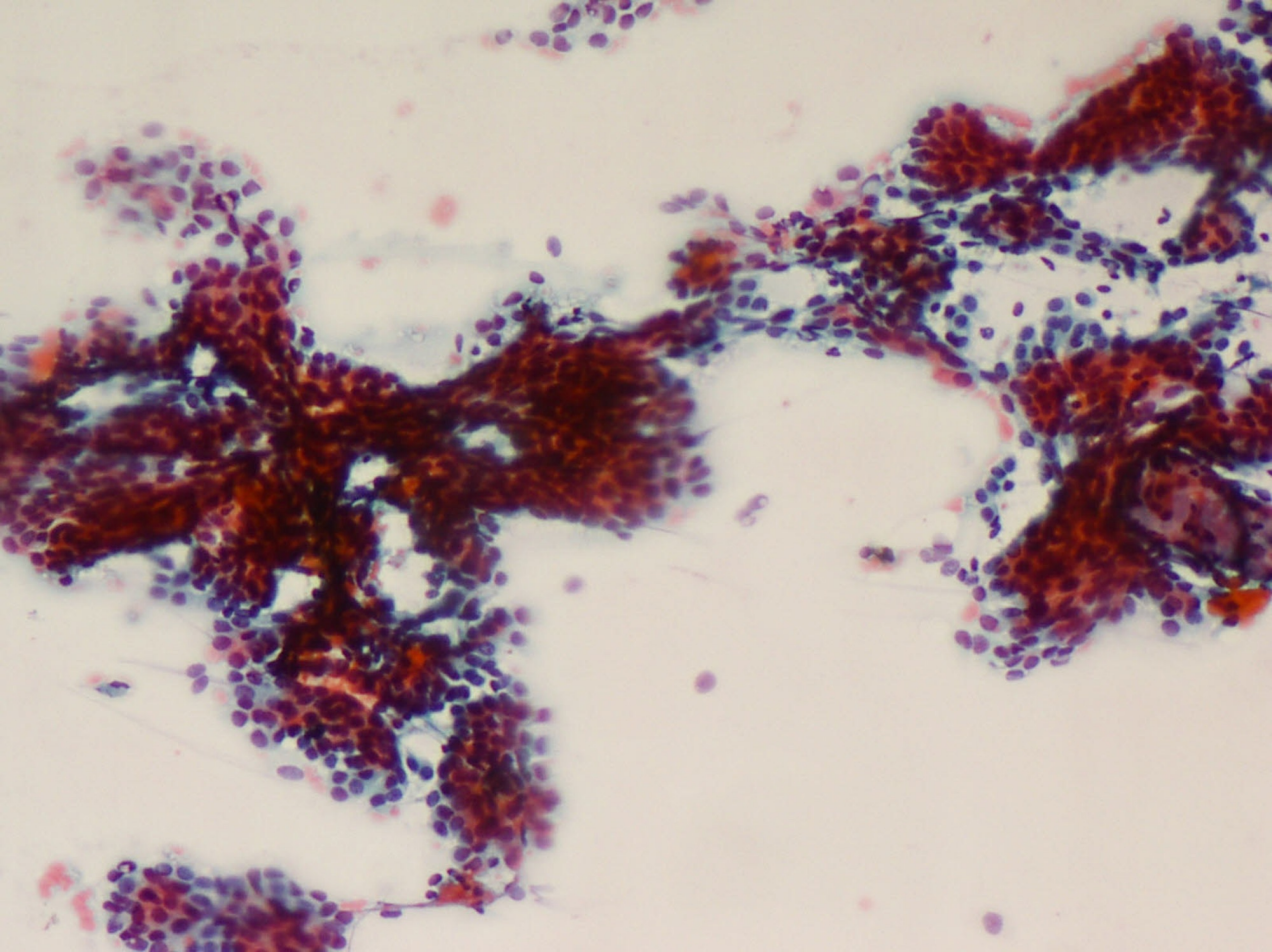
FNAC: Cohesive clusters with branching papillary fronds (PAP x100) PAP: Papanicolaou stain; FNAC: fine-needle aspiration cytology

**Figure 3:**
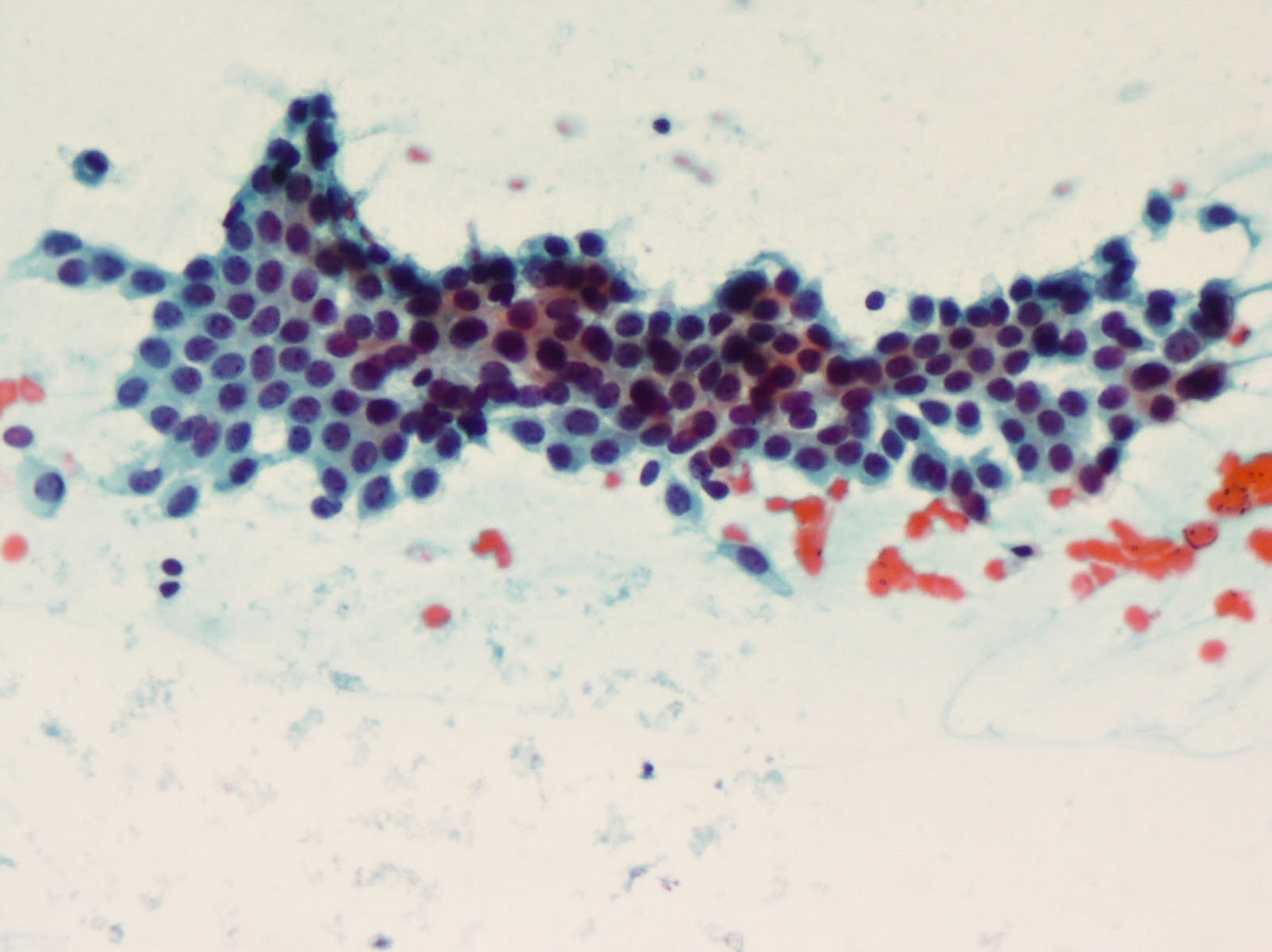
FNAC: Cohesive clusters with branching papillary fronds (PAP x200) PAP: Papanicolaou stain; FNAC: fine-needle aspiration cytology

Similarly, fine needle aspiration cytology (FNAC) showed multiple cohesive clusters of branching papillary fronds suggestive of fibroadenoma (Figures 2-3).

**Figure 4:**
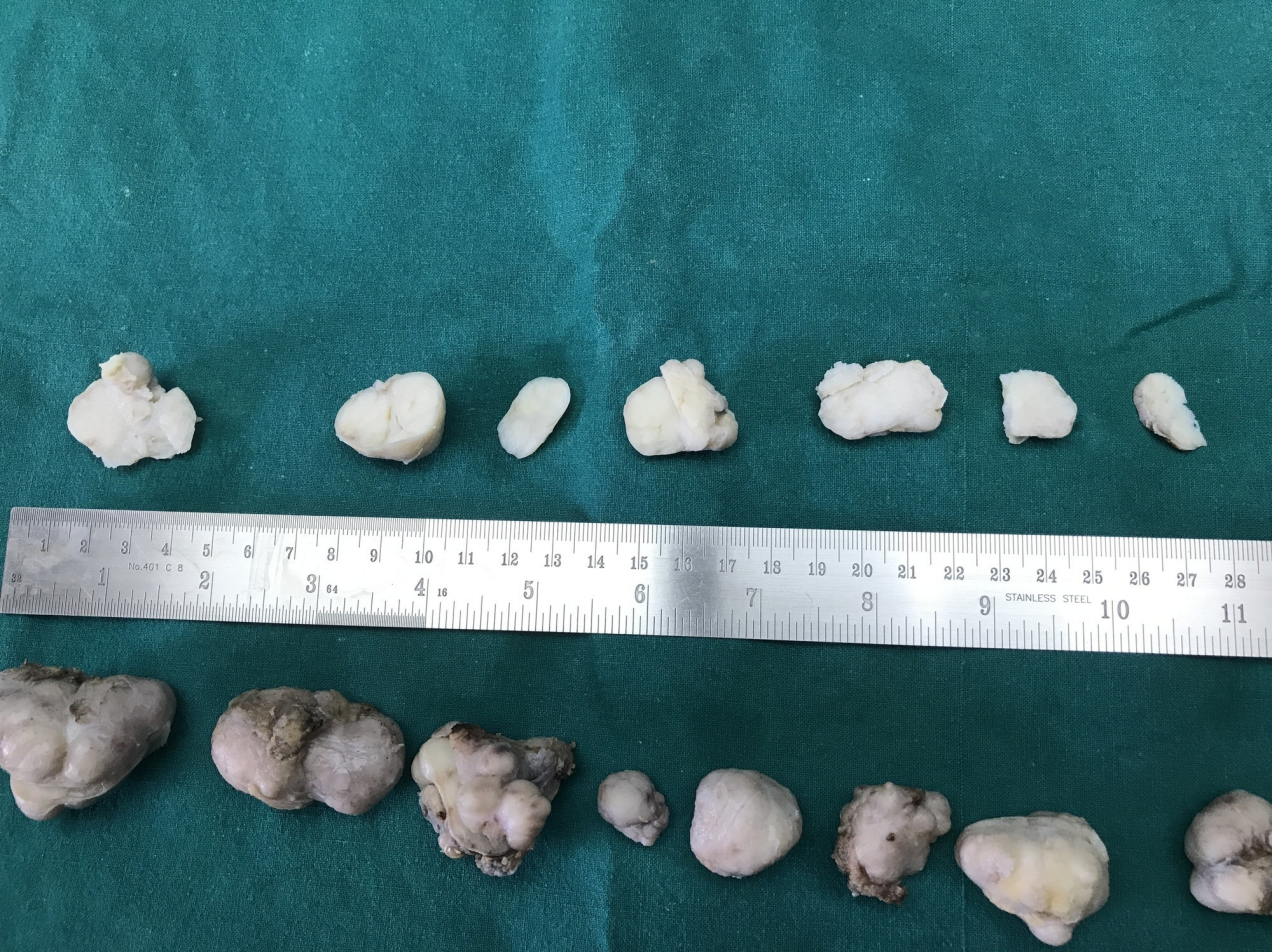
Gross: Multiple, well-circumscribed encapsulated masses

The patient underwent lumpectomy, and the excised specimens were sent for histopathological examination. Gross examination of the excised specimens showed multiple, circumscribed, encapsulated masses with the largest measuring 4x2.5x2 cm. The cut section showed slit-like spaces surrounded by grey-white areas (Figure 4).

**Figure 5:**
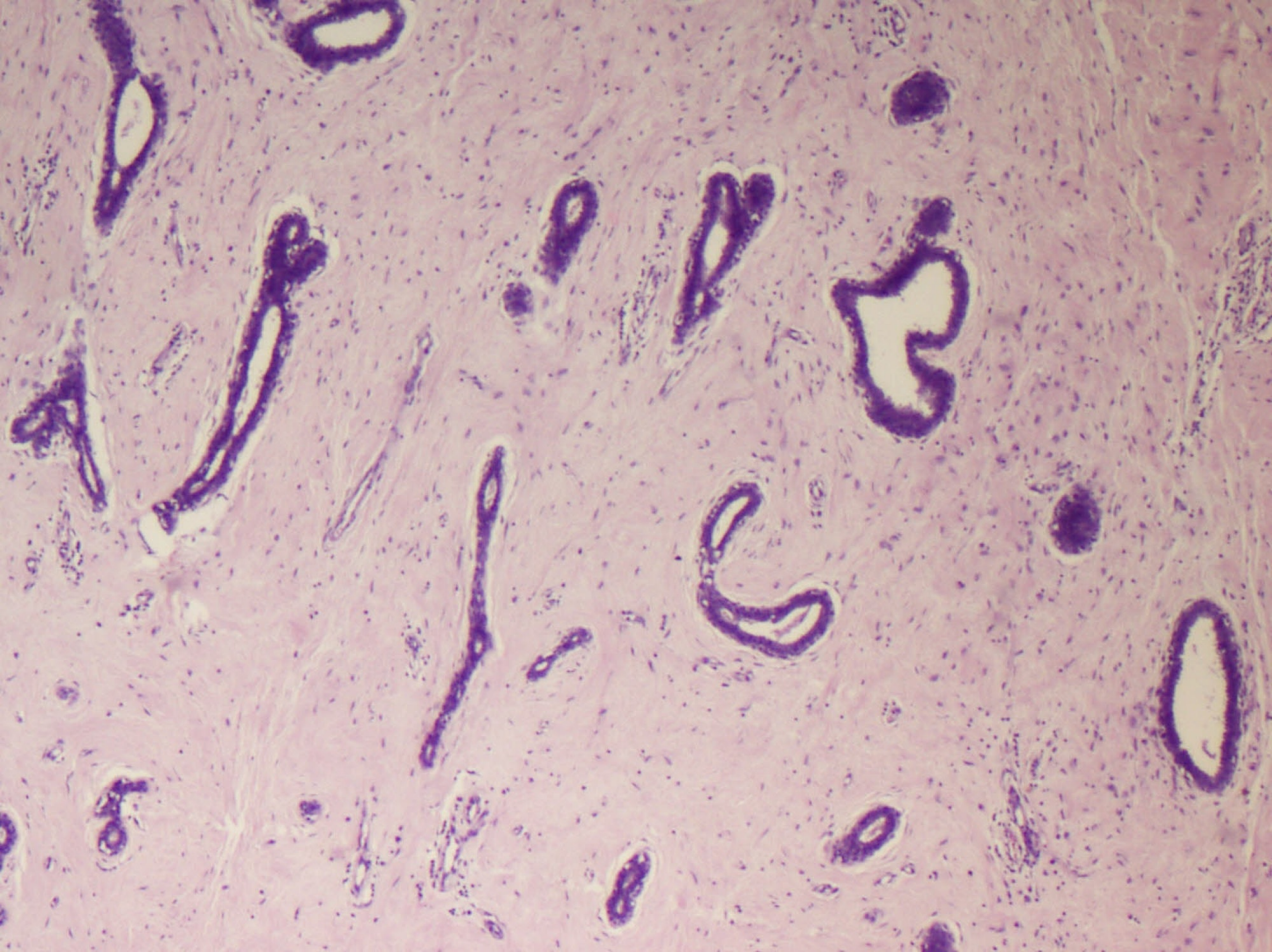
Fibroadenoma with compressed ducts (H&E x100) H&E: hematoxylin and eosin

**Figure 6:**
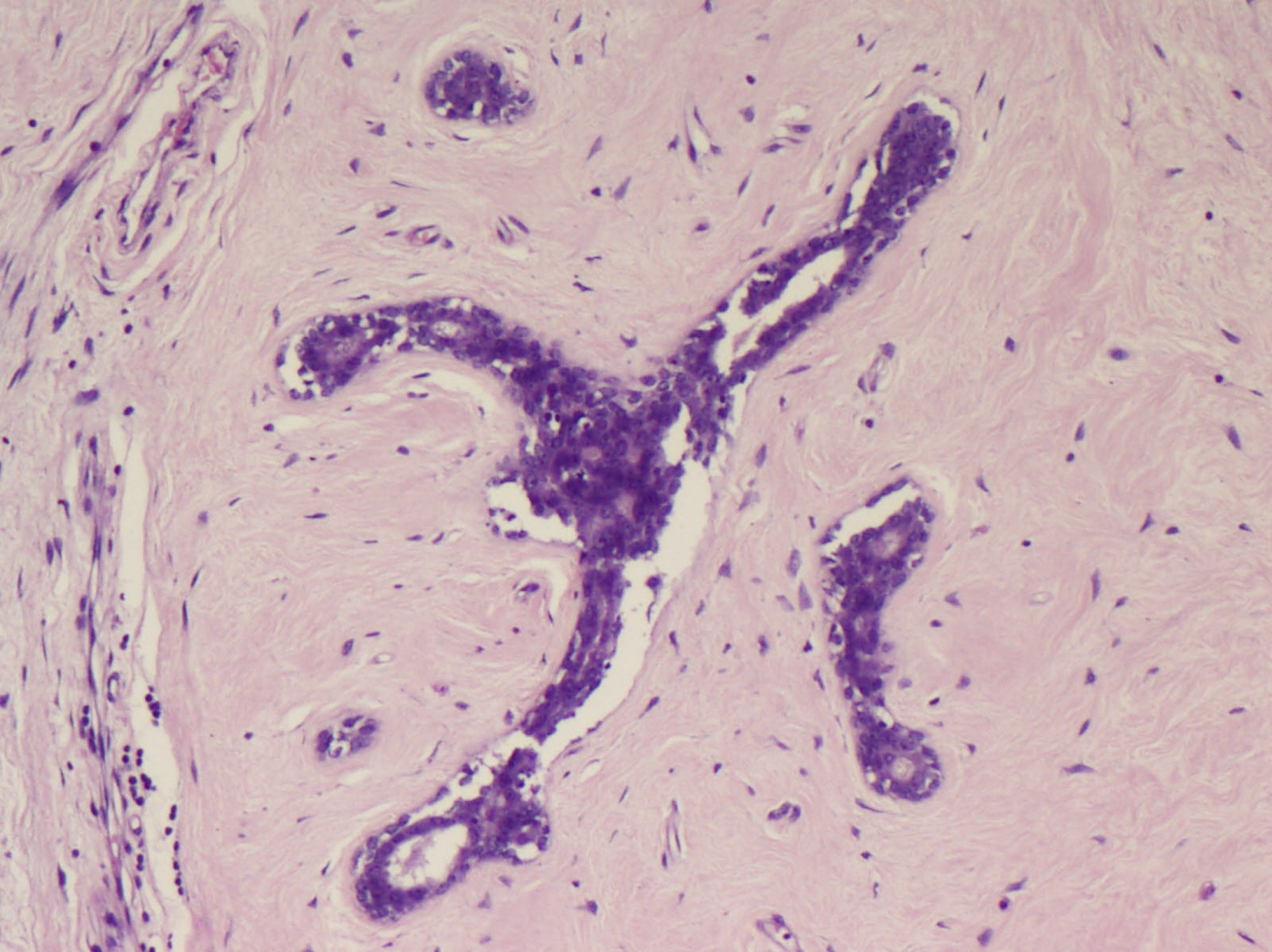
Fibroadenoma with compressed ducts (H&E x200) H&E: hematoxylin and eosin

**Figure 7:**
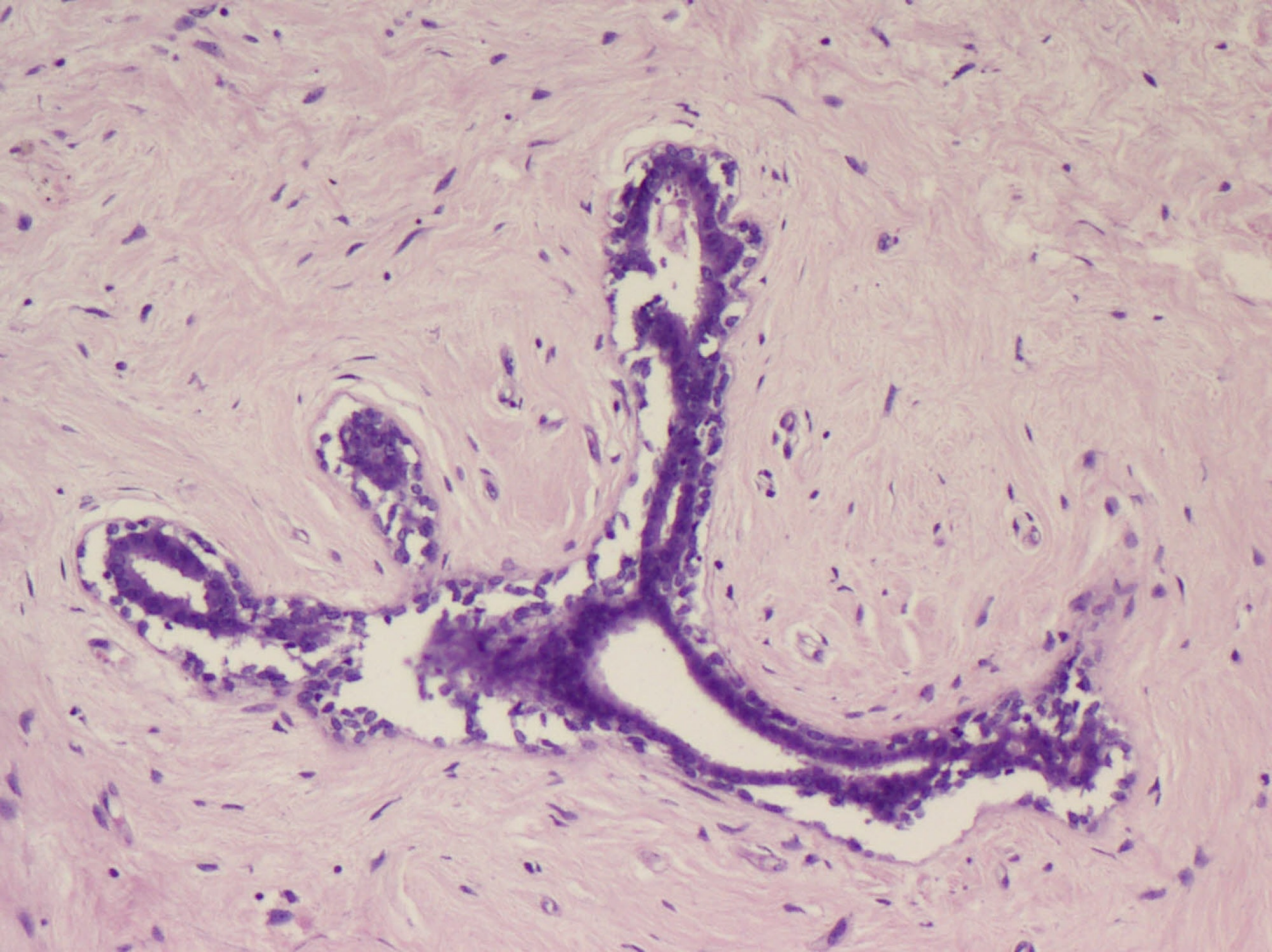
Fibroadenoma with compressed ducts (H&E x200) H&E: hematoxylin and eosin

**Figure 8:**
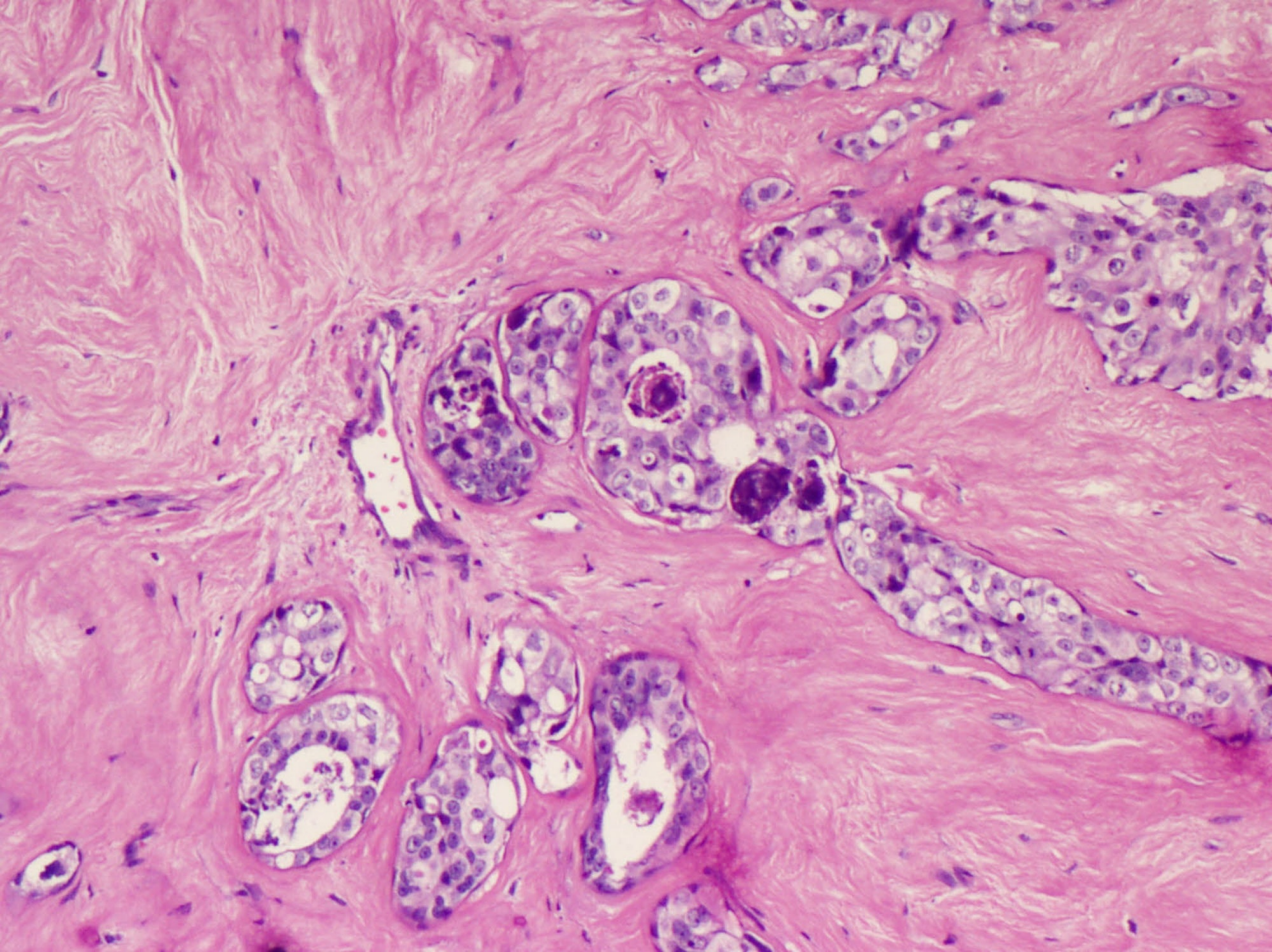
Cellular proliferation with comedo necrosis (H&E x100) H&E: hematoxylin and eosin

**Figure 9:**
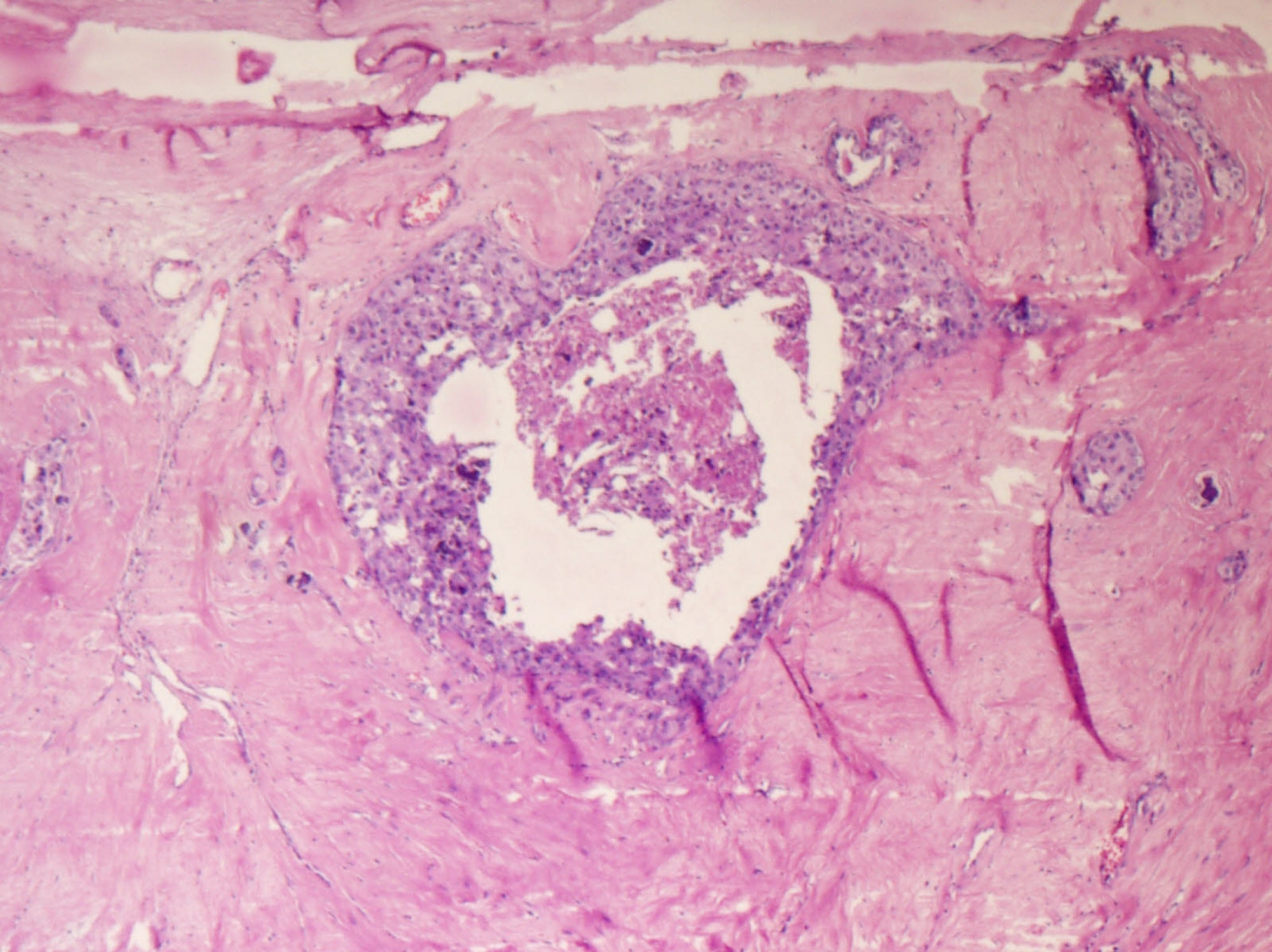
Cellular proliferation with comedo necrosis (H&E x100) H&E: hematoxylin and eosin

**Figure 10:**
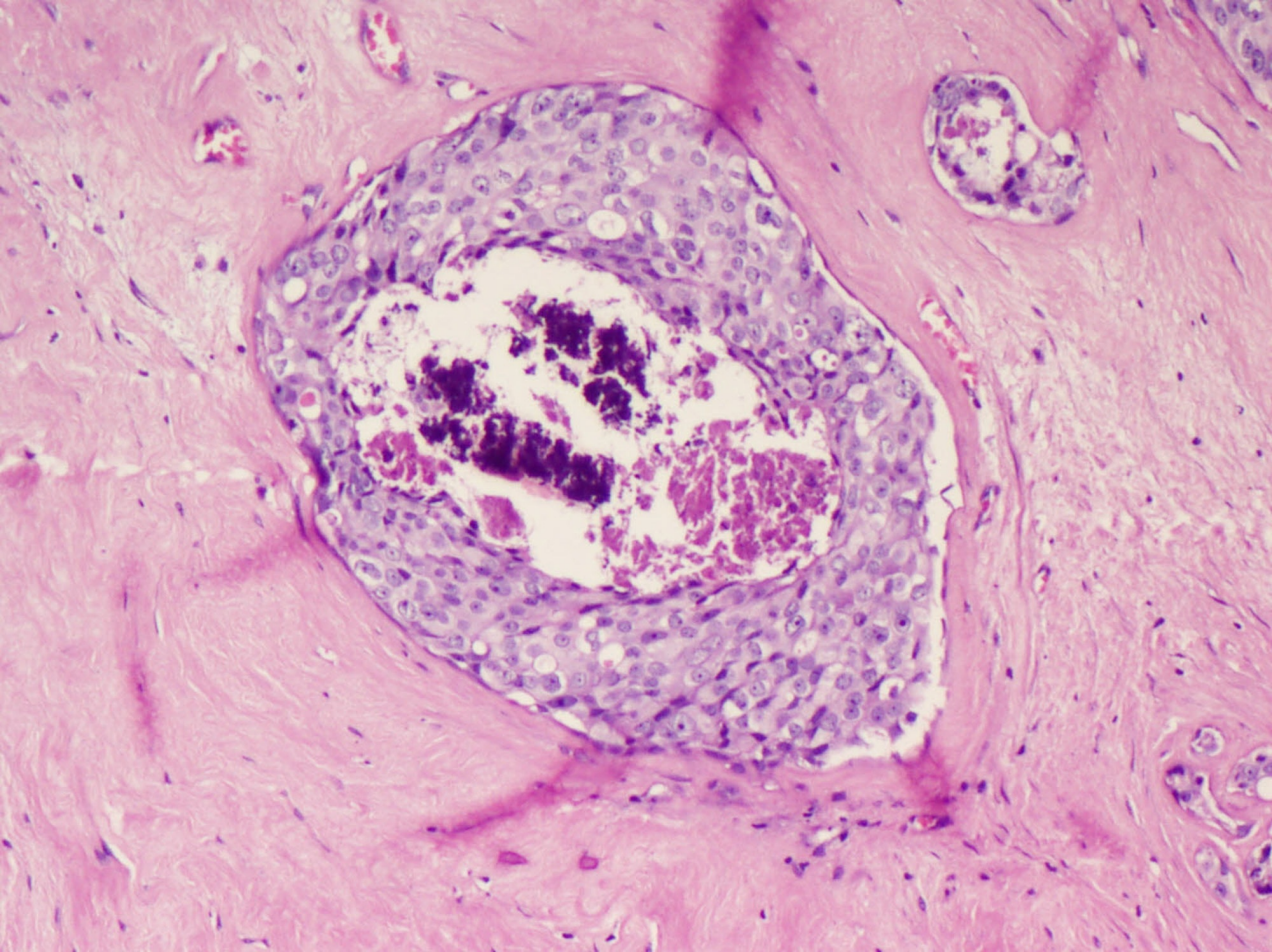
Cellular proliferation with comedo necrosis (H&E x200) H&E: hematoxylin and eosin

Microscopy predominantly showed an encapsulated tumor composed of proliferating acini lined by epithelial and myoepithelial cells, many of which are compressed by fibrous stroma, which is myxoid in areas (Figures 5-7). At the foci, there were areas showing nests of malignant cells having pleomorphic nuclei, prominent nucleoli, and eosinophilic cytoplasm with atypical mitosis, a cribriform pattern, and comedo necrosis, suggestive of ductal carcinoma in situ (Figures 8-10). Stroma surrounding the carcinoma was normal without any evidence of atypical cell invasion.

## Discussion

A fibroadenoma is a common benign tumor of the breast with a peak incidence at the second and third decades of age. A fibroadenoma must be differentiated from benign phyllodes tumor, as carcinomas arising from a phyllodes tumor are also reported in the literature. Fibroadenomas can be classified as simple or complex. Clinically, fibroadenomas present with a lump in the breast, which was the presenting complaint in this case. A preoperative diagnosis of carcinoma arising from these lesions is difficult because of the close resemblance of the presenting features with benign fibroadenoma [3-4]. To evaluate breast lesions, triple tests were done and include clinical examination, USG, and FNAC [1,5].

The characteristic USG features of fibroadenomas include round, hypoechoic lesions with smooth borders and normal surrounding areas while in case of malignancy, the features are irregular shape and margins, hypoechogenicity, and posterior acoustic shadowing [6].

The FNAC findings of fibroadenoma are clusters of branching papillary fronds of benign ductal epithelial cells, myoepithelial cells, and sparse stromal fragments in a fibromyxoid background. While in the case of malignancy, FNAC shows a high nuclear-cytoplasmic ratio (N:C ratio) with pleomorphism and hyperchromatic nuclei.

In the present case, these features were not present on FNAC, therefore, the diagnosis was given as a benign lesion. Because of the limitations of FNAC for selective sampling, the confirmation can only be done by a histopathological examination of the excised specimen. In the present case, the diagnosis was evident only on a histopathological examination.

The diagnostic criteria of DCIS within fibroadenomas require showing at least one of the following findings: (a) intraductal carcinoma focus, seen in the adjacent breast tissue, or (b) intraductal proliferative lesions within fibroadenomas show cancer-characteristic findings, e.g., epithelial necrosis [7].

Definitive treatment consists of surgery with or without radio or chemotherapy. Surgical management depends on the stage at the time of presentation and the presence of axillary or distant metastasis [8]. In the presenting case, excision with breast conservative surgery was done for in-situ carcinoma arising from fibroadenomas. Prognosis is good in cases of early detection.

## Conclusions

This case highlights the rare association of fibroadenoma and carcinoma in situ. In this case, USG and FNAC revealed only multiple fibroadenomas. The carcinoma was detected incidentally on histopathological examination. Therefore, a careful and extensive sampling of the tissue is required to prevent a false negative diagnosis by pathologists.
